# Genome-Wide Identification of Glutathione S-Transferase and Expression Analysis in Response to Anthocyanin Transport in the Flesh of the New Teinturier Grape Germplasm ‘Zhongshan-HongYu’

**DOI:** 10.3390/ijms23147717

**Published:** 2022-07-13

**Authors:** Hui Li, Yaxin Yang, Haoran Li, Wu Wang, Huan Zheng, Jianmin Tao

**Affiliations:** 1 Laboratory of Fruit Tree Biotechnology, College of Horticulture, Nanjing Agricultural University, Nanjing 210095, China; 2017104024@njau.edu.cn (H.L.); 2021104024@njau.edu.cn (Y.Y.); 2021204007@njau.edu.cn (H.L.); zh889111@163.com (H.Z.); 2 Institute of Botany, Jiangsu Province and Chinese Academy of Sciences, Nanjing 210014, China; 2017204015@njau.edu.cn

**Keywords:** GST, genome-wide, gene family, teinturier grape, anthocyanin

## Abstract

Anthocyanins are synthesized in the endoplasmic reticulum and then transported to the vacuole in plants. Glutathione S-transferases (GSTs) are thought to play a key role in anthocyanin transport. To clarify the mechanism of GST genes in the accumulation and transport of anthocyanin in the early fruit stage, we analyzed and characterized the GST family in the flesh of ‘Zhongshan-HongYu’ (ZS-HY) based on the transcriptome. In this study, the 92 GST genes identified through a comprehensive bioinformatics analysis were unevenly present in all chromosomes of grapes, except chromosomes 3, 9 and 10. Through the analysis of the chromosomal location, gene structure, conserved domains, phylogenetic relationships and *cis*-acting elements of GST family genes, the phylogenetic tree divided the GST genes into 9 subfamilies. Eighteen GST genes were screened and identified from grape berries via a transcriptome sequencing analysis, of which 4 belonged to the phi subfamily and 14 to the tau subfamily, and the expression levels of these GST genes were not tissue-specific. The phylogenetic analysis indicated that *VvGST4* was closely related to *PhAN9* and *AtTT19*. This study provides a foundation for the analysis of the GST gene family and insight into the roles of GSTs in grape anthocyanin transport.

## 1. Introduction

Glutathione S-transferases (GSTs) are an ancient protein family that have been found in almost all organisms [1]. The GST protein contains two conserved domains, including the GSH-binding domain (N-terminus) and the substrate-binding domain h-site (C-terminus). However, the members of amino acid sequences show low similarity and the structures of the proteins are remarkably similar [2]. Glutathione S-transferases are characterized by their ability to metabolize toxic compounds and are often thought of as detoxification enzymes caused by the reduced tripeptide glutathione (GSH) conjugation [3]. The GST-mediated cellular detoxification process mainly includes transferase and peroxidase enzymatic activities. When acting as transferases, the conjugates resulting from GSTs unite with the organic molecules’ electrophilic center to mediate CSH and are exported from the cytosol to the vacuole or the apoplast [4,5], but when acting as peroxidases, the GSTs can reduce the lipid peroxides to alcohols [6]. The plant GSTs play roles in cellular metabolism and detoxification in plants and have been intensively studied for herbicide detoxification [7]. In addition to the above functions, the plant GSTs have been demonstrated to be involved in the stress responses caused by pathogen attack and oxidative and heavy metal stresses. The plant secondary metabolites such as anthocyanins and the cellular response to auxins are affected by plant GSTs [8]. The plant GST members are classified into TAU, phi, lambda, theta, zeta, EF1Bγ, DHAR and TCHQD groups [9], and the GSTs involved in anthocyanin transport belong to the unique phi group in plants [10]. Genome-wide analyses of the GST family have been performed in *Arabidopsis thaliana* [11], apple [12], corn [13], citrus [14], pak choi [15], radish [16], tomato [17], hami melon [18] and pepper [19].

Anthocyanins are flavonoid compounds, which are responsible for the color of fruits. The color of the berry is an important trait of the grape. In grapes, two kinds of genes involved in anthocyanin transport, namely a glutathione-S-transferase (GST) and MATE-type transporters, have been characterized [20]. *VviGST4* and *VviGST1* were shown to participate in the accumulation of anthocyanin pigments in Vitis vinifera [21]. Many studies have indicated that the weakening of the GST function could lead to decreased anthocyanin accumulation. The insertion and deletion of GST bases were the reasons for the white color in peach flowers [22]. A similar situation also includes *MdGST* in apple [23] and Riant in peach [24]. *PpGST1* regulates anthocyanin accumulation in peaches through the coordinated interaction with *PpMYB10.1* [25]. *AcGST1* was upregulated by *AcMYBF110* by directly binding to the promoter [26].

Most of the research on grape anthocyanins has focused on the skin, and there are few studies specifically on teinturier table grape anthocyanins. Unlike most anthocyanins that accumulate only in the skin, the teinturier grapes accumulate anthocyanins in both the skin and flesh [27]. In the red flesh variety ‘Alicante Bouschet’, the accumulation of anthocyanins begins in the flesh and moves to the skin [27]. In grape ‘Yan-73’, the anthocyanins are synthesized in the grape flesh and also accumulate in the stem epidermis, leaves and stalks of grapes [28]. The main anthocyanins in the grape skin are malvidin derivatives, while in the flesh they are peonidin derivatives [29]. Peonidin and petunidin derivatives form important components of anthocyanins in Yan-73 flesh. According to our previous research, the main anthocyanins in the ‘Zhongshan-HongYu’ (ZS-HY) flesh are malvide and peonidin derivatives, and their contents are very similar [30]. Unlike in ‘Alicante Bouschet’ and ‘Yan-73’, 52 DDA and 55 DDA (days after anthesis), there was a transition in berry color, and the flesh of ‘ZS-HY’ began to turn red 7–10 days after anthesis [31]. Therefore, it is necessary to study the role of GST genes in the transport of early anthocyanins in the flesh of ‘ZS-HY’.

However, the biological function of GST genes in teinturier grapes remains rare. In this study, we present the GST family member identification, sequence analysis, phylogenetic relationships, chromosomal distribution and collinearity analysis results across species. Furthermore, to better identify the role of GST genes in anthocyanin transport in grapes, we perform an integrated analysis of the transcriptome and genome-wide investigation of grapes. In total, 18 genes are identified in the transcriptome data. The study of GST genes can provide a foundation for the characterization of their function and can promote the selection of candidate genes in teinturier grapes through bio-techniques.

## 2. Results

### 2.1. Identification and Characterization of the GST Family

To identify the members and the characteristic function of the GST family based on the grape genome, the published 61 *Arabidopsis thaliana* GST protein sequences and 52 apple GST protein sequences were used as queries for BLAST and HMM searches and a total of 108 members of GST genes were preliminarily screened. The candidate proteins were further verified by the CDD, Pfam and SMART websites, and 92 GST proteins containing both GST_C and GST_N domains were identified. The 92 GST genes were renamed according to their positions on the chromosomes (except *VvGST1*, *VvGST3* and *VvGST4*).

The molecular characteristics of the GST protein sequences were analyzed (Table S1). The results showed that the amino acid lengths of the GST proteins were between 200 and 424 aa, and the molecular weights were between 22.96 and 48.31 k Da. The maximum and minimum proteins were encoded by *VvGST68* and *VvGST20*, respectively. The isoelectric points were between 4.99 (*VvGST37*) and 9.68 (*VvGST12*). Except for the hydrophilicity indexes of *VvGST30* and *VvGST32*, which were positive, the others were negative, indicating that most of them were hydrophilic proteins, but the degree of hydrophilicity was different. It was predicted that most proteins were located in the cytoplasm, with a few located in the plasma membrane, mitochondria and nucleus.

### 2.2. Phylogenetic Analysis of the GST Gene Family

**Figure 1:**
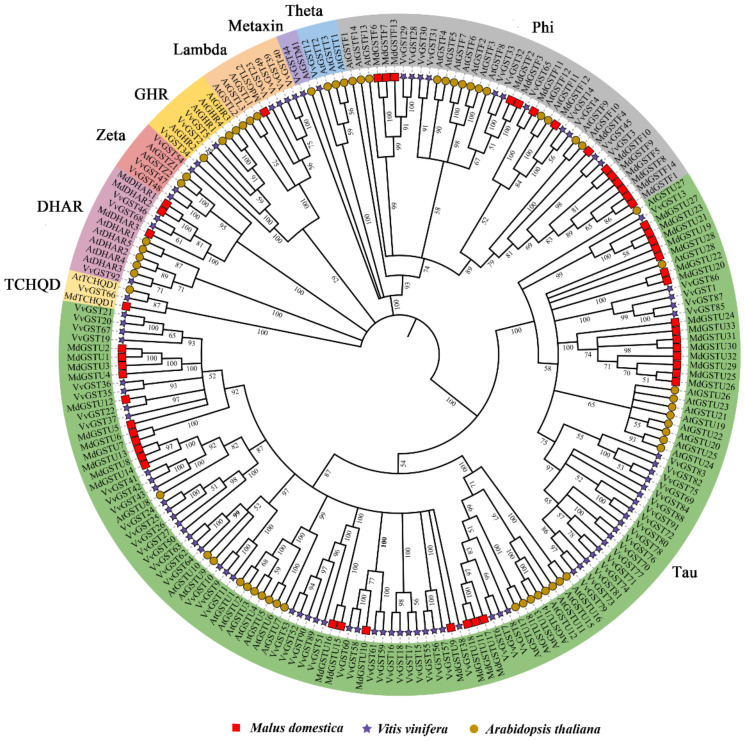
Phylogenetic tree constructed with 92 GST protein sequences with the GST genes from *Arabidopsis thaliana* and *Malus domestica*. Different colors represent different subfamilies of GST genes, different shapes indicate different species. The MEGA X software was used to construct the tree using the neighbor-joining method.

To analyze the phylogenetic relationship of GST proteins, the 92 VvGST proteins, 61 AtGST proteins and 52 MdGST proteins were aligned using MEGA X software and the phylogenetic tree was constructed. The phylogenetic tree analysis showed that the 92 VvGST proteins could be divided into 9 subfamilies (Figure 1). The tau subfamily has the largest number of members, including 64 VvGST proteins, followed by the phi subfamily (11 members), while the Thea, Metaxin and TCHQD subfamilies all have only 1 protein.

### 2.3. Chromosomal Location and Collinearity Analysis of GST Genes

**Figure 2:**
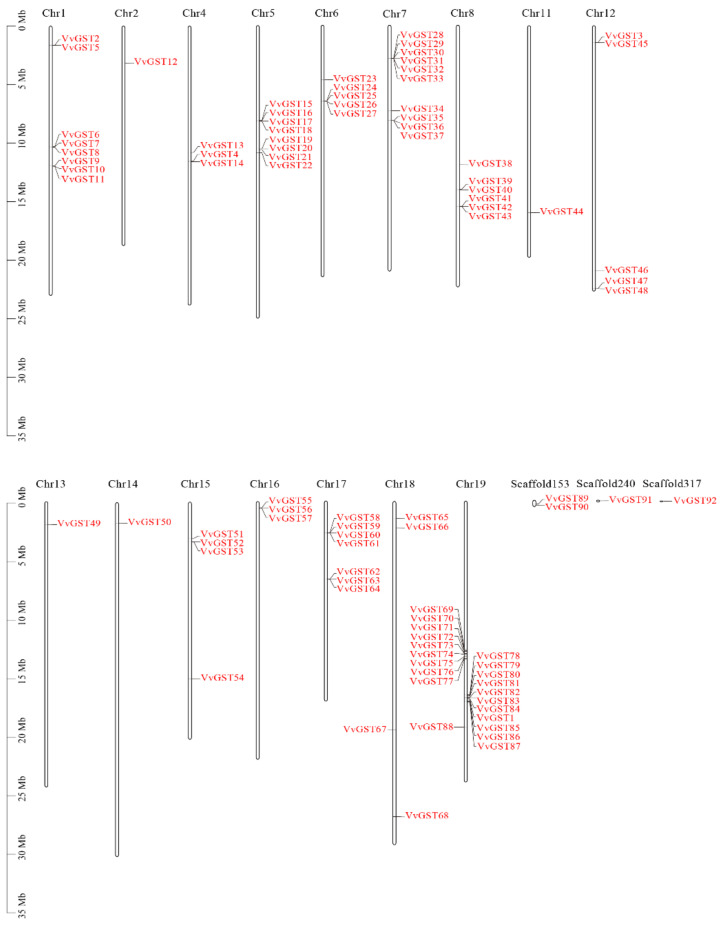
Chromosome localization of grape GST family members.

The chromosome location analysis of VvGST genes based on genome annotation information showed that the 92 VvGST genes were unevenly distributed on 19 chromosomes, of which chromosome 19 had the largest number of members with 21 GST genes, followed by chromosome 7, containing 10 members. However, chromosomes 3, 9 and 10 did not contain any GST genes. *VvGST89*, *VvGST90*, *VvGST91* and *VvGST92* were located on scaffolds, not on any chromosome (Figure 2). This can easily be due to wrong or incomplete genome assembly and limitations of sequencing means.

**Figure 3:**
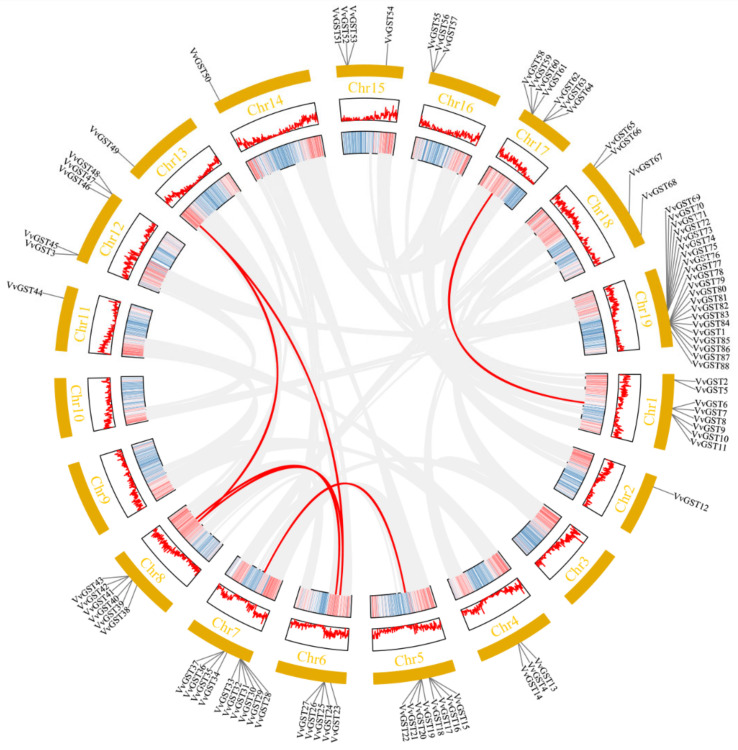
The collinearity analysis of the GST gene family in grape. The GST genes were mapped onto different chromosomes. The red lines indicate segmental duplicated gene pairs of grape GST genes, while the gray lines delineate synteny blocks in the grape genome.

Subsequently, we analyzed the duplication events of the GST genes by using the Multiple Collinearity Scan toolkit (MCScanX). Among the 92 GST genes, 37 members were assigned to tandem duplication, 12 to WGD or segmental duplication, 16 to dispersed duplication and 17 to proximal duplication (Table S2). A total of 32 pairs of duplicated genes were identified in the grape GST family (Figure 3), including 8 pairs of segmental pairs, involving 13 genes. *VvGST23*, *VvGST39* and *VvGST49* were involved in multiple duplication events (Table S3). The other 24 pairs of genes were involved in tandem duplication.

**Figure 4:**
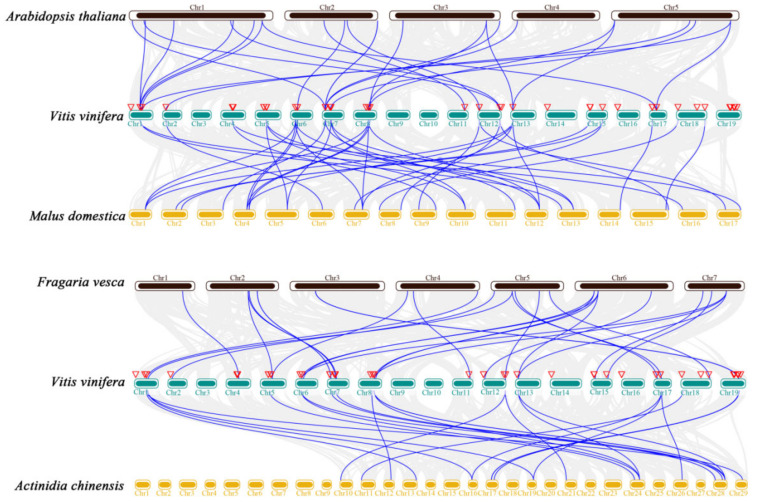
The synteny analysis of the GST genes between grape, *Arabidopsis thaliana*, *Malus domestica*, *Fragaria vesca* and *Actindia chienesis*. The blue lines indicate segmental duplicated gene pairs of grape GST genes with other species, while the gray lines delineate synteny blocks in the grape genome.

To further elucidate the potential evolutionary mechanism of the VvGST gene family, TBtools was used to screen homologous genes in grape, *Arabidopsis thaliana*, apple, *Fragaria vesca* and *Actindia chienesis* (Figure 4 and Table S4). In total, 30 pairs of grape and strawberry homologous genes and 26 pairs of grape and kiwifruit homologous genes were identified. A total of 26 pairs of homologous genes in grape and *Arabidopsis thaliana* were identified, involving 13 VvGST genes and 40 pairs of grape and apple homologous genes involved in 20 VvGST genes, which may have been due to the smaller phylogenetic distance between grape and apple.

### 2.4. Gene Structure and Motif Analysis of GST Genes

**Figure 5:**
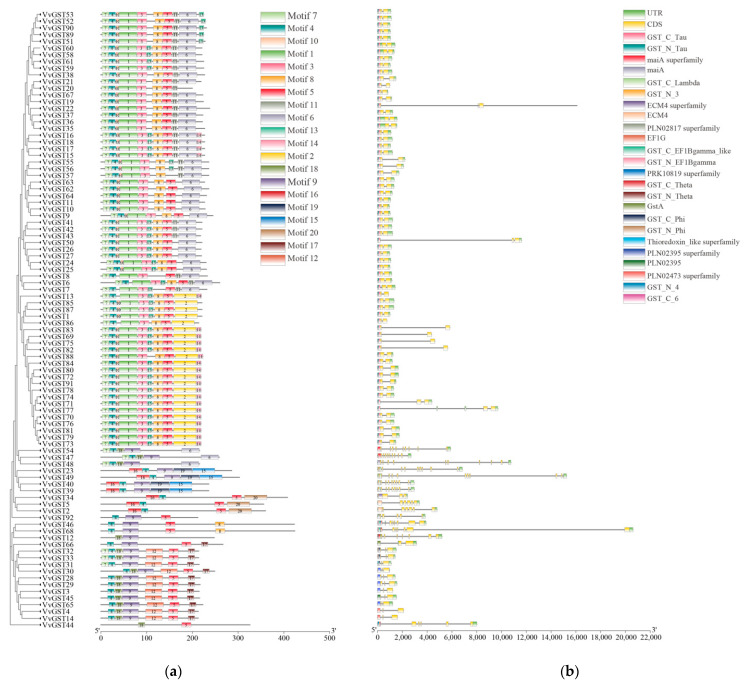
Protein motifs and DNA structures of GST genes family in grapes. (**a**) The protein motifs in the GST members. Different colored boxes indicate different motifs. (**b**) The DNA structures of the GST gene family in grapes. Different colored boxes delineate different structures.

To further understand the composition of grape GST proteins, a motif analysis was carried out by using the MEME online website, and 20 conserved motifs were identified (Figure 5a). In different subfamilies, there were differences in the compositions of the conserved motifs of the GST proteins, which may reveal the different functions of GST genes. For example, motifs 1, 3, 4 and 7 were present in all members of the tau subfamily, whereas motif 6 was only present in the zeta subgroup and motif 20 was only detected in the GHR subfamily. Motifs 1, 4 and 7 were annotated as GST_N, while motifs 2, 3, 4, 6 and 8 were annotated as GST_C. Motif 12 only existed in the phi subfamily and was annotated as GST_C. Motifs 15 and 19 were C-terminal domains unique to the lambda subfamily. The MEME analysis showed that each subgroup had similar motif characteristics, which further supported the clustering of the GST family in the phylogenetic tree.

TBtools was used to visualize the exon and intron structures of the 92 GST genes (Figure 5b). The results showed that the conserved domains of the GST family members in the same subfamily were almost identical, but there were great differences among different subfamilies. The numbers of exons of GST genes were between 2 and 10. The genes with two exons belonged to the tau subfamily, and all members of the phi subfamily contain only three exons. The subfamilies with the largest numbers of exons were zeta (*VvGST47*, *VvGST48*) and lambda (*VvGST29*, *VvGST49*). These results showed that the exon-intron structure of the same class of genes was relatively conservative and closely related to the evolution of the GST family.

### 2.5. Identification of cis-Acting Elements in the Promoter Region of GST Genes

**Figure 6:**
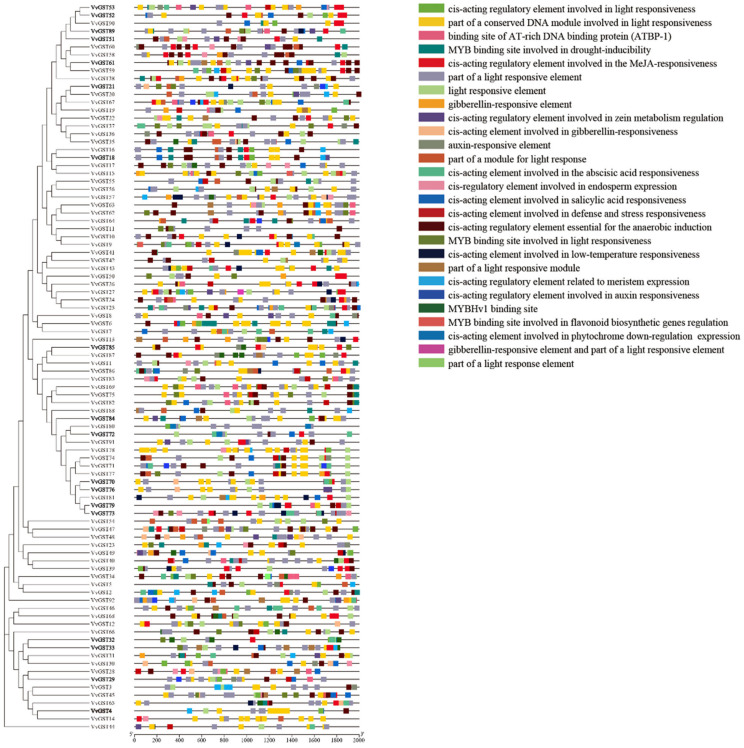
Prediction of *cis*-acting elements in the GST promoters. The 2000 bp sequence upstream of the promoters was analyzed and different regulatory elements are shown in different colors.

The grape genome sequence and GFF file were downloaded from NCBI. We obtained the genes 2000 bp upstream of the start site of genes by using the TBtools sequence/fasta extraction function, and then analyzed their *cis*-elements to understand the gene function and regulation mechanism of GST genes. The results showed a large number of *cis*-acting elements in the promoter region upstream of the genes, which can be combined with transcription factors to regulate various biological processes in plants. Plant CARE was used to analyze the promoter of the GST genes, and 47 *cis*-acting elements were predicted, including ABRE, Box 4, CAT-box, CGTCA-motif, GT1-motif, GATA-motif, MRE and MBS. These *cis*-acting elements can respond to plant biological and abiotic stresses, plant hormones, light and low temperature (Figure 6), indicating that GST genes may have diverse functions. Some binding sites of MYB transcription factors related to anthocyanin synthesis were also identified in these promoters, which may play an important role in grape anthocyanin accumulation. Based on the previous studies, we compared the *cis*-acting elements in the promoter region of the GST genes among grape, *Arabidopsis thaliana* [11] and apple [32]. The result showed that the types of *cis*-acting elements had high similarities among the three species. The number of light response elements was the largest, followed by MeJA, abscisic acid and other response elements. There were MYB binding sites in these three species, which may be related to the function of GST genes in anthocyanin transport (Table S5). Based on this result, we speculated that the GST gene family has multiple and similar functions among different species.

### 2.6. Phenotypic Characterization of the Flesh of ‘ZS-HY’ and ‘ZC’

**Figure 7:**
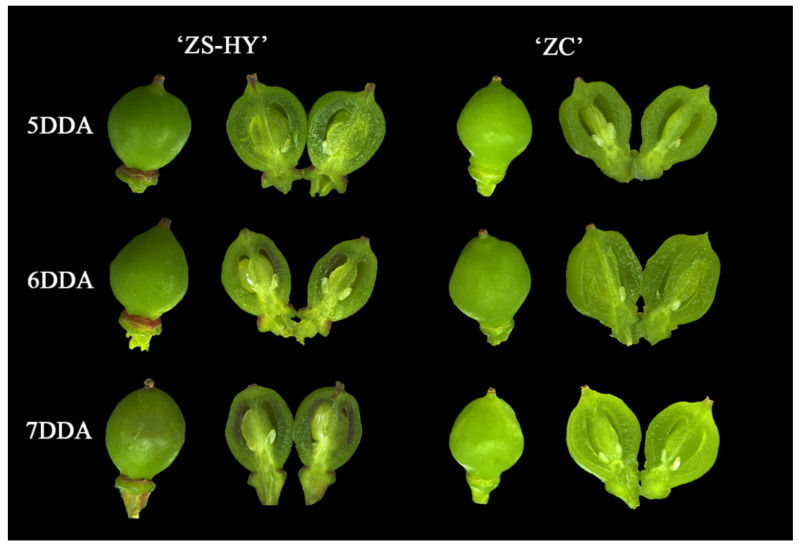
Change in appearance of the flesh color in grapes for 3 consecutive days.

According to our observations, the berries of ‘ZS-HY’ change in color 5–7 days after anthesis, and the color of the flesh turns fully red in the later stage of fruit development (Figure 7). The composition of anthocyanins in the flesh of ‘ZS-HY’ mainly includes malvidin, cyanidin and peonidin derivatives (Table 1). With the deepening of the red color in the flesh, the content and types of anthocyanins increased. However, no accumulation of anthocyanins was detected in ‘ZC’.

### 2.7. Analysis of Transcriptome Data and the Expression Analysis of GST Genes in Different Tissues

**Figure 8:**
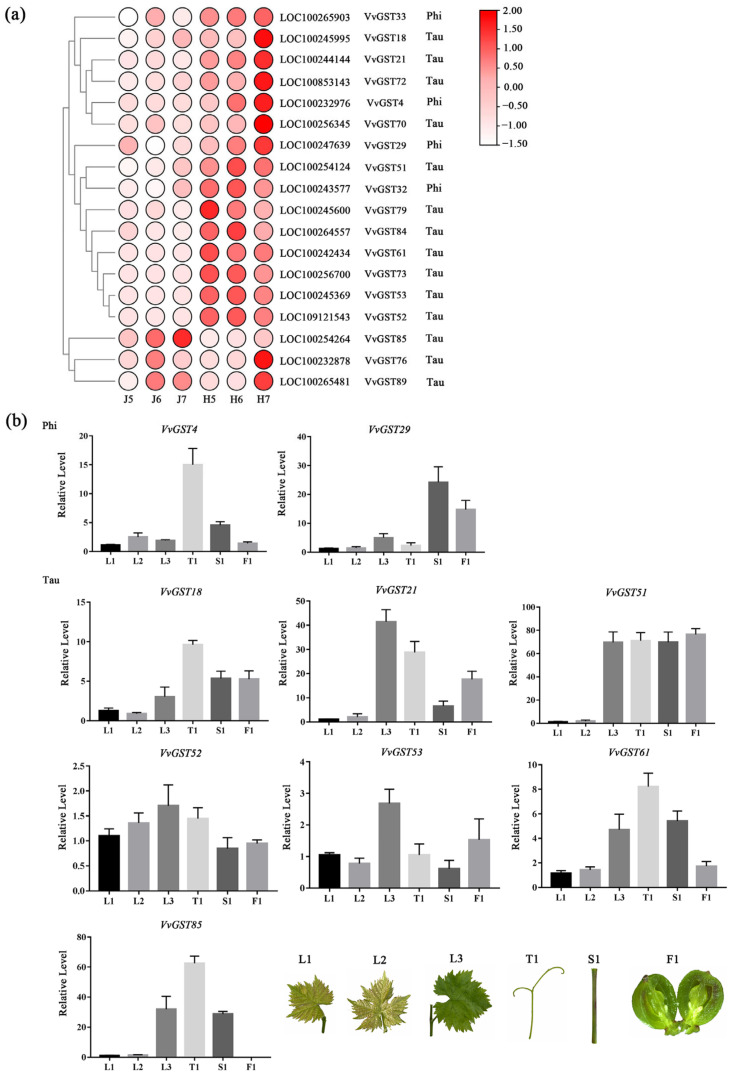
Heat map of the expression patterns of GST genes and relative expression of GST genes in six tissues. (**a**) Heat map of the expression patterns of the GST genes. The expression levels of genes are shown using different colors from white (−1.5) to red (2), with the values representing the log2 FPKM values. A redder color indicates a higher gene expression level. (**b**) Relative expression of GST genes in six tissues. The left y-axis represents the relative gene expression levels analyzed by qRT-PCR.

To further verify the GST genes related to anthocyanin transport in the early stage of grape development, we performed a transcriptome sequencing analysis of ‘ZS-HY’ of the flesh for three consecutive days and a clustering analysis among genes based on gene expression patterns. The results showed that only 18 GST genes were detected during the process of early anthocyanin accumulation in ‘ZS-HY’, and 2 subfamily types were identified, which included phi and tau. As a result (Figure 8a and Table S6), only 4 GST genes (*VvGST4*, *VvGST29*, *VvGST32*, *VvGST33*) belong to the phi subfamily and the rest belong to the tau subfamily. Most of genes had a low expression in ‘Zico’ (ZC) but a higher expression in ‘ZS-HY’. All of these genes could be divided into three groups based on their expression patterns: (a) the expression of genes increased with the accumulation of color in the flesh and reached the highest level at 7DDA, such as *VvGST33*, *VvGST18*, *VvGST21*, *VvGST72*, *VvGST4*, *VvGST70* and *VvGST29*; (b) the expression of genes increased with the accumulation of color in the flesh but did not reach the highest level at 7 DDA, such as *VvGST51*, *VvGST32*, *VvGST79*, *VvGST84*, *VvGST61*, *VvGST73*, *VvGST53* and *VvGST52*; (c) the genes were more highly expressed in ‘ZC’ than in ‘ZS-HY’, such as *VvGST85*, *VvGST76* and *VvGST89*, indicating that these genes may be related to growth and development rather than anthocyanin transport.

At the same time, to investigate the role of the GST genes, qRT-PCR was used to analyze the expression patterns of 9 GST genes in different tissues, including the leaves in three stages of growth and development, the stems, the tendrils and the red flesh of ‘ZS-HY’. Most of the GST genes were highly expressed in the leaves at the L3 stage, the tendrils and the stems. For the expression patterns of the GST genes and sequence specificity in the RNA-seq data, *VvGST61*, *VvGST4*, *VvGST21*, *VvGST18*, *VvGST29*, *VvGST85*, *VvGST52*, *VvGST51* and *VvGST53* were selected for verification. The *VvGST4* gene with the most significant difference in expression in the flesh reached the highest expression level in the tendrils. *VvGST51* was highly expressed in almost all tissues except the leaves at the L1 and L2 stages. The expression of *VvGST85* was the lowest in the flesh but was higher in the tendrils, stems and leaves at the L3 stage, indicating that it may play a stronger role in the growth and development of ‘ZS-HY’ than in anthocyanin transport. In contrast, *VvGST51*, the sole member of the tau subfamily, was highly expressed in the flesh of ‘ZS-HY’, which indicates its function in anthocyanin transport (Figure 8b and Table S7).

### 2.8. Analysis of GST Genes Evolutionary Relationships in Transcriptome Data

**Figure 9:**
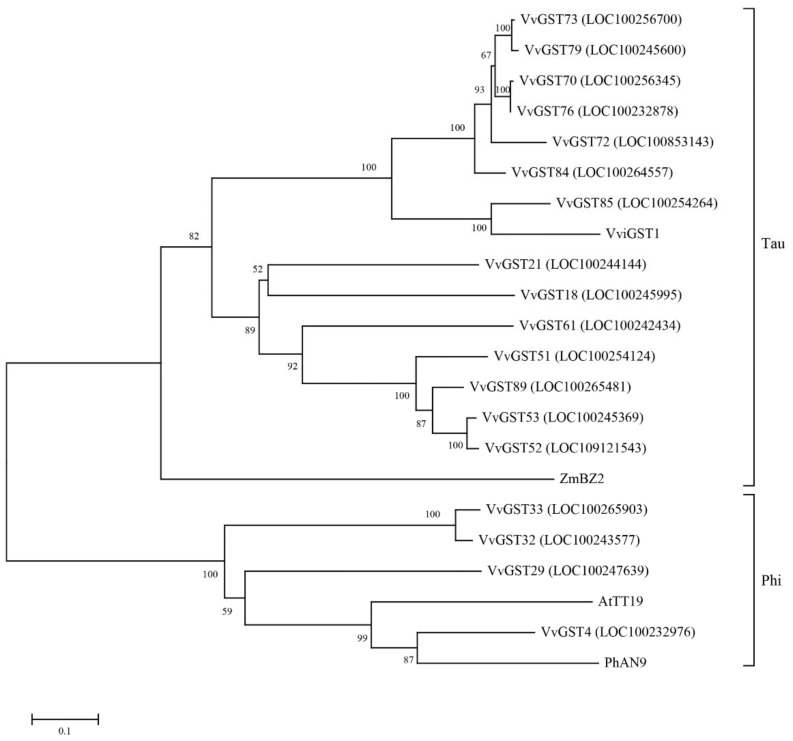
Phylogenetic relationship between 18 GST genes and the PhAN9, AtTT19, ZmBZ2 and VviGST1 proteins. The tree was constructed according to the neighbor-joining method in MEGA X, and the Poisson model, partial deletion and 1000 bootstrap replicates were used.

To compare the genetic relationship between the GST genes in the grape transcriptome and GST family members involved in anthocyanin transport in *Arabidopsis thaliana* ((*AtTT19*) AED92398), *Zea mays* ((*ZmBZ2*) AAA50245), *Vitis vinifera* ((*VviGST1*) AAN85826) and *Petunia hybrida* ((*PhAN9*) CAA68993), the Muscle algorithm in MEGA X software was used for multiple sequence alignment to construct the phylogenetic tree. In the phylogenetic tree, *VvGST4* was homologous with *AtTT19* and *PhAN9*, and *VvGST85* was homologous with *VviGST1*, but no gene was homologous with *ZmBZ2* (Figure 9).

### 2.9. qRT-PCR Verification of Transcriptome Data

**Figure 10:**
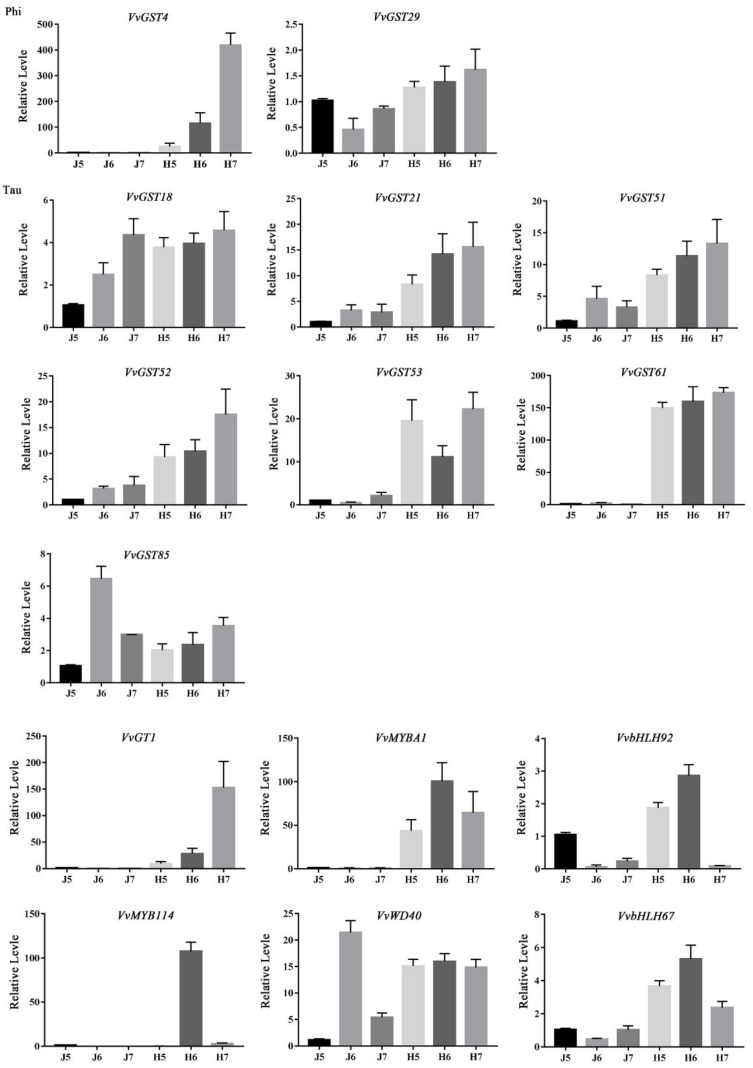
Verification of relative expression levels of genes in RNA-seq by qRT-PCR. The left y-axis represents the relative gene expression levels analyzed by qRT-PCR.

To confirm the accuracy of the transcriptome data and the expression patterns of candidate GST genes in the flesh of ‘ZS-HY’, we selected 15 genes for qRT-PCR analysis. The RNA-Seq and qRT-PCR results were basically in conformity (Figure 10 and Table S7).

## 3. Discussion

The secondary metabolites in plants form a protective complex, which can protect them from herbivores and pathogens. The complex that the cells produce can be toxic itself [33]. Anthocyanins are synthesized in the endoplasmic reticulum and localized to the vacuole [34]. The GST genes in the plants have been proven to be related to anthocyanin transport and they have been characterized. In maize, the mutated gene Bronze-2 that is involved in anthocyanin transport leads to a bronze pigment in cells, which causes the improper accumulation of anthocyanins [35]. *AN9* plays an important role in anthocyanin export to the vacuole in petunia and performs an effect analogous to that of *Bz2* in maize [34]. *AtTT19* is required for the transport of anthocyanins in *Arabidopsis thaliana* [10]. These genes except *ZmBz2* belong to the phi subfamily; *ZmBz2* was classified into the tau subfamily [35,36]. However, the GST genes involved in anthocyanin transportation are still unclarified in teinturier ‘ZS-HY’ grapes. In this study, we performed a genome-wide identification of GST genes in the grape genome, based on phylogenetic, structural and evolutionary characteristics. At the same time, this was combined with transcriptome and qRT-PCR approaches to screen out key genes involved in anthocyanin transportation.

To date, 61, 59, 61, 139, 49, 130 and 52 GSTs have been found in *Arabidopsis thaliana* [37], rice [38], citrus [14], lichi [39], Cucumis melo [40], strawberry [41] and apple [32], respectively. We identified 205 GST genes from grape, *Arabidopsis thaliana* and *Malus domestica* and divided them into 9 subfamilies based on their sequence characteristics and phylogenetic relatedness (Figure 1). They were unevenly present in all chromosomes except for chromosomes 3, 9 and 10 in grapes (Figure 2). Through the prediction of subcellular localization of GST proteins in grapes, we found that most VvGST proteins were localized in the cytoplasm, with few localizations in the mitochondria, plasma membrane or nucleus (Table S1). In addition, we also analyzed the collinearity between grape GST genes and *Arabidopsis thaliana*, apple, strawberry and kiwifruit. Species with a relatively close evolutionary relationship, such as apple and strawberry, have more collinear gene pairs, with as many as 40 collinear gene pairs with apple (Figure 4).

Previous studies have demonstrated that the expansion of gene families is due to gene duplication events such as tandem duplication, segmental duplication and whole-genome duplication during evolution [42,43]. The reason for the enlargement of the GST gene family in plants is mainly due to the expansion of the tau and phi subfamilies. In our study, the expansion of the VvGST family was found to be mainly driven by tandem duplication, as 37 out of 91 VvGSTs were aligned to tandem duplication events (Figure 3). Therefore, tandem replication events may be the main driver for VvGST expansion. Differences in the structures of gene exons and introns can lead to differences in gene structure [44,45]. The presence of introns provides evolutionary protein diversity through increased exon shuffling and alternative splicing [46]. The members of the same group are more likely to have similar genetic structures. Our gene structure (motif and intron–exon) analysis indicated that the same classes of GST genes were similar in structure (Figure 5).

Interestingly, based on the transcriptome analysis, the 18 GST genes identified in our transcriptome data all mapped to the tau and phi subfamilies. Among these 18 candidate GST genes, those belonging to the phi subfamily were only from chromosomes 4 (*VvGST4*) and 7 (*VvGST29*, *VvGST32*, *VvGST33*), and the rest of GST genes from the tau subfamily were located in chromosomes 5 (*VvGST18*, *VvGST21*), 15 (*VvGST51*, *VvGST52*, *VvGST53*), 17 (*VvGST61*) and 19 (*VvGST70*, *VvGST72*, *VvGST73*, *VvGST76*, *VvGST79*, *VvGST84*, *VvGST85*), as well as the scaffold (*VvGST89*) (Figure 2 and Figure 9).

In grapes, the ability of *VvGTS1* and *VvGST4* to transport anthocyanins has been confirmed, and these genes complement anthocyanin transport in Bronze-2-deficient corn kernels [47]. *VvGST4* as a candidate gene for anthocyanin transport was identified in the berry of muscadine grapes [48]. Notably, the expression level of *VvGST4* in the phi subfamily was significantly upregulated, with a fold change of more than 50, increasing with the increase in anthocyanin content and reaching the highest level at 7 DDA in our RNA-seq data (Figure 8a and Table S6). Compared with ‘ZC’, the expression difference in GST genes in the flesh of ‘ZS-HY’ was more obvious, indicating that GST genes play an important role in the transport of anthocyanins in the flesh. However, the tissue-specific analysis of GST genes in ‘ZS-HY’ indicated that the effect of the GST genes on ‘ZS-HY’ may include not only anthocyanin transport, but also tissue growth and development (Figure 8b). The result showed that GST genes are not tissue-specific in ‘ZS-HY’, and many genes had high-level expression in the stems, leaves and tendrils, implying the potential function of these gens in stem, leaf and tendril development. The phylogenetic tree analysis showed that *VvGST4* was most closely related to *PhAN9* in *Petunia hybrida*, followed by *AtTT19* in *Arabidopsis thaliana* (Figure 9). These results suggest that *VvGST4* may play an important role in the process of teinturier grape anthocyanin transport. The remaining GST genes also play a role in anthocyanin transport, but their efficacy may be less than that of *VvGST4*. Furthermore, GST genes related to anthocyanin transport and anthocyanin biosynthesis genes are known to be coordinately expressed, and the expression pattern is consistent with anthocyanin accumulation. The expression patterns of *VvGST4* and the anthocyanin pathway genes *VvUFGT* and *VvMYBA1* are similar in grapes, suggesting possible co-regulation between them [47]. In our results, the expression patterns of the GST genes were similar to those of the genes involved in anthocyanin biosynthesis (Figure 10 and Table S7).

Numerous researchers have reported that the expression of GST genes is affected by some external and internal factors. *AtTT19* was upregulated due to the overexpression of *PAP1* (a R2R3 MYB gene) for anthocyanin accumulation in *Arabidopsis thaliana* [49]. The work on kiwifruit revealed that the expression of *AcGST1* was promoted by *AcMYB110* and could complement the anthocyanin-free phenotype of *AtTT19* [26]. Similar results were obtained for strawberry [50], apple [51], litchi [39] and peach [25]. In addition to this, some studies have shown that the transcription of *LcGST4* in litchi was significantly induced by exogenous ABA hormone treatment and light [39]. The transcript level of *PpGST1* was provoked by UV irradiation [52]. In this study, some hormone-responsive, light-responsive and MYB-binding site elements were predicted in the promoter of GST genes (Figure 6), indicating that the GST genes might be regulated by both external and internal factors.

These findings provide a functional predictive analysis of the GST genes for anthocyanin transport in ‘ZS-HY’, while functional assays are required for further validation. At the same time, the regulation of anthocyanins by GST genes in grape may involve complex network regulation, which requires further study.

## 4. Materials and Methods

### 4.1. Plant Materials

‘ZC’ and a new teinturier grape germplasm ‘ZS-HY’ were used as plant materials, grafted in an experimental field at Nanjing Agricultural University, Jiangsu, China. According to our observations, the berries of ‘ZS-HY’ change in color for 5–7 days after anthesis, and the color of the flesh turns fully red in the later stage of fruit development. A similar situation appeared in the later stage of fruit development for ‘ZC’, and their phenological periods are similar. Berries with similar growth rates were sampled for three consecutive days, denoted as 5 DDA, 6 DDA and 7 DDA, corresponding to 5 days, 6 days and 7 days after anthesis (named H5, H6, H7), respectively, while ‘ZC’ was collected as a control (named J5, J6 and J7, respectively). All berries were sampled from the same location of each cluster of three independent trees. Here, 15–20 berries were collected and the skin was separated from the flesh for use in the RNA-seq process and the determination of the anthocyanin content. Two biological replicates were used at each stage for the treatment and control.

To verify the specificity of GST gene expression, we collected three different growth and development stages (named L1, L2 and L3, respectively) of the leaves, tendrils (named T1), stems (named S1) and red flesh (named F1); all samples were randomly sampled from 3 vines at the same time. Each tree was used as a biological replicate, and 3 biological replicates were collected for each sampling. Here, 15–20 berries were collected and the peel was separated from the flesh, with only the flesh being kept and all tissues being stored at −80 °C for further experiments.

### 4.2. Identification of the GST Family

The protein and nucleotide sequences of grapes were downloaded from NCBI (https://www.NCBI.nlm.nih.gov/; accessed on 10 April 2022). The GST proteins of *Arabidopsis thaliana* and *Malus domestica* were obtained from the *Arabidopsis* Information Resource (https://www.arabidopsis.org/index.jsp; accessed on 10 April 2022) and Phytozome (https://Phytozome-next.jgi.doe.gov/; accessed on 10 April 2022), respectively. The AtGSTs and MdGSTs were used as queries to search for VvGST candidate genes with a cutoff E-value ≤ 1 × 10^−5^ using Blastp. HMMER was used to search against grape protein sequences with hidden Markov model profiles (PF00043 and PF02798). The candidate genes obtained by Blastp and HMM were merged to remove redundant genes. For the candidate genes obtained via preliminary identification, the conserved domain prediction software programs CDD [53] (https://www.ncbi.nlm.nih.gov/Structure/cdd/wrpsb.cgi; accessed on 20 April 2022), Pfam (https://pfam.xfam.org/search; accessed on 20 April 2022) and SMART [54] (https://SMART.embl-heidelberg.de; accessed on 20 April 2022) were used to ensure that they contained both GST_C and GST_N domains [9].

### 4.3. Phylogenetic Analysis

The GST protein sequences of grape, *Arabidopsis thaliana* and apple were aligned using the Muscle program. Then, MEGA X software was used to construct the phylogenetic tree via the neighbor-joining method [55]. The Poisson model, partial deletion and bootstrap process replicated 1000 times were selected. The iTOL online website (https://itol.embl.de/; accessed on 21 April 2022) was used to visualize the phylogenetic tree.

### 4.4. Chromosomal Location and Collinearity Analysis of GST

The chromosome location was completed based on the genome data of grapes in the NCBI database (https://www.ncbi.nlm.nih.gov/; accessed on 10 April 2022). The location map was drawn by TBtools. The MCScanX was used to analyze duplicate pairs and duplicate types [56], and the results were visualized using Circos in TBtools [57].

### 4.5. Gene Structure and Domain, and Motif Analysis

The structural information of the GST genes was obtained from a GFF file, and TBtools was used to visualize the conserved domain and exon-intron structure of the GST genes. The conserved motifs of GST proteins were analyzed using the online analysis program MEME version 5.4.1 (https://meme-suite.org/meme/tools/meme; accessed on 30 April 2022). The matching length range of the conserved motifs was 6~50 aa, and the number of conserved motifs was 20. TBtools was used to visualize the conserved motifs based on the output file from MEME version 5.4.1 [58].

### 4.6. Analysis of cis-Acting Elements in GST Gene Promoters

The 2000 bp upstream sequences of the VvGST genes’ CDS were extracted by TBtools, and Plant CARE (http://bioinformatics.psb.ugent.be/webtools/plantcare/; accessed on 18 April 2022) was used to predict the *cis*-acting elements in the promoter region of the VvGST gene family [59], then the simplified results were visualized using TBTools.

### 4.7. Measurement of Anthocyanin Content

The extraction of anthocyanin was carried out according to the previous description [30] and measured using high-performance liquid chromatography (HPLC), which was performed based on the previously reported protocol [60]. The mobile phases of aqueous 1% formic acid and methanol were employed for analysis at a flow rate of 0.2 mL·min^−1^. A 5 uL shot volume of each sample was used and chromatograms were detected at 525 nm by using LC–MS system (G2-XS QT, Waters). Anthocyanins were quantified at 525 nm as malvidin 3-glucoside chloride equivalents. Experiments were repeated three time with two independent samples.

### 4.8. RNA-Seq Data Analysis of ‘ZS-HY’

To study the expression profiles of the 92 GST genes involved in the transport of anthocyanin during early growth and development in the flesh of ‘ZS-HY’, we analyzed transcriptome data in the samples of ‘ZC’ and ‘ZS-HY’ at 5-7 DDA. RNA sequencing libraries were constructed using Illumina HiSeqTM 4000. The heat map of the GST genes was constructed using TBtools.

### 4.9. Validation of Gene Expression

In total, 15 genes were selected for qRT-PCR using the ABI 7300 system (ABI, Los Angeles, CA, USA). The primer design was performed with Beacon Designer 7 software and the primers used are listed in Table S7. *VvActin* from grapes was used as the internal reference gene. The experiments were conducted with three biological replicates, and the data analysis of each gene was performed using the 2^−ΔΔct^ method. The qRT-PCR statistical analysis was performed with the software GraphPad prism V7.0.0 (Motulsky, Graphpad software, Los Angeles, CA, USA).

## 5. Conclusions

The synthetic pathway of anthocyanin has been well studied, but anthocyanin transport is also an indispensable step in the process of anthocyanin delivery to the vacuole for storage. To reveal the function of GST genes in teinturier ‘ZS-HY’ grapes, we identified a total of 92 GST genes and classified them into 9 subtribes. Subsequently, we performed transcriptome sequencing and screened out 18 GST genes, which were classified only into phi and tau. It is worth noting that *VvGST4* performed particularly well among the 18 GST genes. This study provides a comprehensive understanding of the GST family in ‘ZS-HY’, especially the ability to participate in anthocyanin transport, and these results provide a systematic analysis of the grape GST gene family and provide candidate GST genes for anthocyanin accumulation. The color of grapes has high commercial value as an important appearance quality. Producing brightly colored grapes is also an important goal of grape breeding. Our research can guide the molecular breeding of grapes and provide useful red flesh resources to improve grape hybrid breeding. This has important implications for the development of new grape varieties with enhanced anthocyanins and consumer appeal.

## Figures and Tables

**Table 1:** Composition of anthocyanins in the flesh of ‘ZS-HY’. Note: ‘----’ indicates that the substance was not detected.

Component Name	Content (μg/g^−1^) FW					
	J5	J6	J7	H5	H6	H7
Malvidin 3-O-glucoside	----	----	----	0.65	0.67	1.21
Malvidin 3-O-(6″-p-coumaroyl-glucoside)	----	----	----	0.65	0.70	----
Peonidin 3-O-glucoside	----	----	----	0.62	0.73	1.33
Malvidin 3-O-(6″-caffeoyl-glucoside)	----	----	----	0.60	0.63	0.90
Peonidin 3-O-(6″-caffeoyl-glucoside)	----	----	----	----	0.61	0.72
Peonidin 3-O-feruloyl-glucoside	----	----	----	----	----	1.49
Cyanidin 3-O-glucoside	----	----	----	----	----	0.65
Petunidin 3-O-(6″-p-coumaroyl-glucoside)	----	----	----	----	----	0.61
Cyanidin 3-O-(6″-p-coumaroyl-glucoside)	----	----	----	----	----	0.60
Total anthocyanins	0	0	0	2.52	3.34	7.52

## Data Availability

All data generated during this study are included in this published article. The raw data for RNA-Seq data generated in this study are available in the SRA of NCBI (https://www.ncbi.nlm.nih.gov/sra, accessed on 2 April 2022) repository under the submission number SUB11272164.

## Supplementary Materials

The following supporting information can be downloaded at: https://www.mdpi.com/article/10.3390/ijms23147717/s1.
